# Is Word Order Responsive to Morphology? Disentangling Cause and Effect in Morphosyntactic Change in Five Western European Languages

**DOI:** 10.3390/e27010053

**Published:** 2025-01-09

**Authors:** Julie Nijs, Freek Van de Velde, Hubert Cuyckens

**Affiliations:** Department of Linguistics, KU Leuven, 3000 Leuven, Belgium; freek.vandevelde@kuleuven.be (F.V.d.V.); hubert.cuyckens@kuleuven.be (H.C.)

**Keywords:** Kolmogorov complexity, Granger causality, morphological complexity, word order rigidity, diachronic linguistics

## Abstract

This study examines the relationship between morphological complexity and word order rigidity, addressing a gap in the literature regarding causality in linguistic changes. While prior research suggests that the loss of inflectional morphology correlates with the adoption of fixed word order, this study shifts the focus from correlation to causation. By employing Kolmogorov complexity as a measure of linguistic complexity alongside Granger Causality to examine causal relationships, we analyzed data from Germanic and Romance languages over time. Our findings indicate that changes in morphological complexity are statistically more likely to cause shifts in word order rigidity than vice versa. The causal asymmetry is robustly borne out in Dutch and German, though waveringly in English, as well as in French and Italian. Nowhere, however, is the asymmetry reversed. Together, these results can be interpreted as supporting the idea that a decline in morphological complexity causally precedes a rise in syntactic complexity, though further investigation into the underlying factors contributing to the differing trends across languages is needed.

## 1. Introduction

Most Indo-European languages, especially the Germanic and the Romance languages, have transitioned from more ‘synthetic’ structures, where grammatical relationships are expressed through inflections, such as case endings on nouns or verbal affixation, to more ‘analytic’ structures, where grammatical relationships are conveyed primarily through word order and function words, such as articles, prepositions, and auxiliaries [1,2,3,4,5,6]. The loss of bound morphology, as a strategy for the expression of morphosyntactic relationships, can be expected to be compensated by word order. Specifically, the existence of a case system tends to inversely correlate with fixed word order, and the deterioration of the verbal morphology can be compensated by auxiliaries and, again, word order. In West Germanic languages, the various verbal positions, viz. the difference between V1, V2, and V-final, signal distinctions in the domain of mood and illocution. This trade-off between morphology and word order is well-known [7,8] and has been established on the basis of corpus data [9,10,11,12], as well as in experimental studies [13,14].

It is a moot point among linguists to determine how the various changes are chronologically and causally related. One perspective argues that the loss of inflectional morphology, such as case endings, necessitated the development of a fixed word order (see ‘configurationality’ in [15,16]) to preserve clarity and comprehension in sentences. According to this view, as languages lost their inflections, speakers relied more on word order to convey grammatical relationships [17,18]. Another perspective suggests that the rise of a fixed word order might have contributed to the loss of inflectional morphology. As certain word orders became more prevalent and entrenched, the need for explicit morphological markers diminished, leading to their eventual erosion [19]. A third viewpoint questions the existence of a direct causal relationship between the two phenomena. Instead, it posits that the decline of inflectional morphology and the emergence of fixed word order could be parallel developments influenced by other language-internal factors such as discourse strategies that aim at rhetorical effectiveness rather than grammatical efficiency [20].

While linguists have always had a strong interest in *how* languages change, they have also given due attention to the (internal) *causes* of language change [21]. Still, the field of linguistics has long been somewhat reluctant to explore causality [22]. When one assumes the knock-on effects of several internal factors, linguists insist one should adduce evidence that goes beyond a simple post hoc ergo propter hoc explanation. In this respect, the temporal co-existence of two historical processes does not necessarily imply that these processes are causally related, even when this relation is highly plausible. This is also well-known in correlational studies: correlation does not imply causation. More recently, significant efforts have been made to explore causation in linguistics, and more specifically the relation between morphology and word order, using methods from adjacent fields of diachronic studies [12,23,24]. These studies provide evidence that morphological changes are indeed the chronological and causal precursors of changes in word order. What has not yet been explored in terms of causality is the relationship between morphological complexity and word order rigidity, which is the focus of the study at hand.

Following [23,24], we used a statistical technique borrowed from economics, namely Granger Causality, to examine the (potentially causal) relationship between morphological complexity and word order rigidity. Rosemeyer and Van de Velde (2021) [24] analyzed a specific aspect of morphosyntax: the relation between word order and clefting in Brazilian Portuguese. For the dependent variable, Moscoso del Prado Martín (2014) [23] used a more ‘holistic’ metric, namely Shannon entropy [25], to look at inflectional diversity (i.e., inflectional entropy) and syntactic diversity (i.e., entropy of the parse tree) in Icelandic. Our study can be seen as expanding upon these two previous studies, by both broadening the scope of the study and shifting the focus from the relation between inflectional morphology and word order to morphological complexity and word order rigidity (as a proxy for syntactic complexity). We combined Granger Causality (see Section 2.2) with Kolmogorov complexity (see Section 2.1 and Section 2.4), an information-theoretic notion that can be used for measuring linguistic complexity [26]. By using Kolmogorov complexity, we avoided having to zoom in on a specific domain within the morphosyntax, and we could instead assess the aggregate morphological complexity and word order rigidity. Both techniques have very specific data requirements: Granger Causality requires uninterrupted time series, while Kolmogorov complexity works best with comparable texts (see Section 2.3). Good data are hard to come by, but with some leeway in the text collections, we were able to examine six different corpora of five different large languages spoken (and written) in Western Europe: Dutch, English, German, Italian, and French, with enough historical depth in their text records to venture into diachronic terrain.

## 2. Data and Methods

### 2.1. Assessing Linguistic Complexity with Kolmogorov Complexity

The study of linguistic complexity is riddled with a variety of terminological and methodological issues. However, two principal concepts of complexity can generally be distinguished: absolute complexity and relative complexity [27,28,29]. **Absolute complexity** approaches complexity as independent of, and unrelated to, any specific language user. It is concerned with the inherent features or phenomena of a linguistic system (e.g., the morphological markers for plural). From an information-theoretic perspective, it relates to the length of the shortest possible description of a given linguistic feature or phenomenon [30]. As it focusses on the inherent complexity of a linguistic system, independent of any agent or user, absolute complexity can be viewed as an ‘objective’ measure. For instance, absolute complexity may be interested in which languages employ more or less complex strategies in specific linguistic domains. **Relative complexity**, on the other hand, adopts a ‘subjective’ perspective, being agent-related and dependent on the experience of language users. This approach defines complexity in terms of the cost, processing effort, or acquisition difficulty as perceived by, and relative to, a language user, such as a speaker, hearer, or learner [27,28]. Typically, the language user referred to in discussions of relative complexity is the second-language learner. Consequently, relative complexity is often equated with the difficulty experienced in second-language acquisition [31,32,33,34]. As an example, relative complexity is, among other things, interested in whether linguistic optionality (i.e., the choice between two morphosyntactic variants) causes production difficulty for the speaker.

Information theory is a branch of applied mathematics and computer science that deals with the quantification, transmission, storage, and processing of information. The main goal of information theory is to measure and analyze the amount of information contained in a message or signal. It provides a mathematical framework assessing how efficiently information can be transmitted through a communication channel and how much redundancy or noise might be present in the data. Information theory was developed by Claude Shannon in the 1940s [25] and has since become a fundamental concept in various fields, including communication systems, cryptography, data compression, and more recently, linguistics.

**Kolmogorov complexity** [35] measures the intrinsic complexity or information content of a data sequence in terms of the length of the shortest possible algorithm required to generate that particular sequence. In essence, Kolmogorov complexity quantifies the compressibility of data; data with low complexity can be compressed efficiently, while data with high complexity cannot be compressed much. The application of Kolmogorov complexity to linguistic complexity was initiated by Juola (1998) [36]. Subsequent investigations delving into this area have been conducted by Bane [37], Juola [38], Sadeniemi et al. [39], Ehret and Szmrecsanyi [40], Ehret [26] and Nijs, Van de Velde and Cuyckens [41].

Kolmogorov complexity calculates the complexity of texts in an unsupervised manner, by exploiting the (ir)regularity and redundancy of linguistic surface structures. In essence, it gauges the repetition and recurrence of orthographic character sequences within a given text. The complexity of a text sample is defined by the length of the shortest possible description that fully represents that sample. When comparing the complexity of two texts, the goal is to describe them as concisely as possible. The text for which the most concise description is the longest is considered the most complex, whereas the text for which the most concise description is the shortest is deemed the least complex. More reducibility means less complexity. For example, the shortest description of the sequence *abababababab* would be ‘ab’*4, while the shortest description of a sequence like *abhirfemholp* would be ‘abhirfemholp’ (i.e., *abhirfemholp* cannot be further reduced). As the most concise description of the sequence *abababababab* is shorter than the most concise description of *abhirfemholp*, the sequence *abababababab* is less complex. A high-complexity sequence consists of random data that requires a lengthy algorithm to reproduce, while a low-complexity sequence has a clear and repetitive pattern that can be represented by a concise algorithm. In a similar way, natural language is characterized by repetition that can be exploited to arrive at the shortest possible description of a text; examples include frequently occurring N-grams such as the Dutch *ik ben geweest* (I have been) or plural suffixes -*en* and -*s* in the Dutch *plant-en* (plant-plural) and Dutch *vogel-s* (bird-plural). While Kolmogorov complexity is a counterintuitive notion to most scholars of language as it completely ignores semantics, it has been proven to be a workable metric of complexity in various fields of applied mathematics.

Unfortunately, Kolmogorov complexity is strictly speaking incomputable because of the Halting Problem [42]. The Halting Problem is concerned with figuring out whether a given program will eventually finish its task (halt) or will keep running forever. Alan Turing showed that there is no general algorithm (set of instructions) that can determine whether any arbitrary program will halt or run forever. The Halting Problem demonstrates the existence of undecidable problems in computation. This implies that there is no algorithm to determine the shortest possible program that generates any given string of data. In other words, there is no general algorithm that can always provide the shortest possible description for all possible inputs.

Since Kolmogorov complexity relies on finding the shortest program to describe data, the undecidability of the Halting Problem implies that it is unfeasible to measure Kolmogorov complexity for all possible inputs. Though Kolmogorov complexity fundamentally remains a theoretical ideal, there are often practical workarounds to approximate Kolmogorov complexity in specific cases, such as assessing the morphological and syntactic complexity of texts. One common procedure involves using data compression algorithms, which achieve compression by finding patterns and redundancies in data and represent it as concisely as possible. Data compression techniques, such as gzip, which was also used in our study, are effective at reducing the size of data by concisely encoding recurring patterns. The size of the compressed data can serve as an approximation of its Kolmogorov complexity.

### 2.2. Determining Causality with Granger Causality

In this study, we wanted to test the causal precedence between changes in morphological complexity and word order rigidity by utilizing a statistical technique known as Granger Causality [43,44], initially developed and used in the field of economics. **Granger Causality** allows us to determine the causal precedence between two time series, denoted as A and B. Specifically, the technique assesses the added value of using prior values of time series B to predict time series A above and beyond the capacity of predicting time series A by prior values of itself, i.e., pure ‘autoregression’. It then tries to do the reverse, using prior values of A to predict B on top of B’s own prior values. Granger Causality is established when an asymmetry arises and only one of the comparisons of the two nested models comes out as significant. The underlying idea is that if time series A causally influences time series B, the dynamics of A contain information that can help reduce the prediction errors (residuals) in B.

As an example, Granger Causality analysis can help assess whether changes in interest rates (A) causally influence housing prices (B) or vice versa. If using prior values of interest rates significantly improves the prediction of housing prices, beyond what housing prices’ own past values can predict, then Granger Causality from A to B is established. On the other hand, if prior housing prices do not significantly improve the prediction of interest rates, there would be no Granger Causality from B to A. This asymmetry would suggest that fluctuations in interest rates influence housing prices, but not the other way around, highlighting the predictive power of A’s dynamics in explaining B.

Granger Causality thus capitalizes on the temporal relationship between two time series, where the first series can be considered a Granger-cause of the second series if changes in the second series frequently follow corresponding changes in the first series. However, it should be noted that Granger Causality alone does not provide definitive proof of causation. Its primary function is to exploit chronological asymmetry to rule out causation in the opposite direction. It does not entirely eliminate the possibility that the two time series may be coincidentally correlated, although this risk is mitigated by incorporating *p*-values to assess the correlation. Another limitation of Granger Causality is that it does not exclude a scenario where trend A Granger-causes trend B because both trends are actually influenced by a deeper trend C (what is known as a ‘confounder’), and B happens to exhibit a delayed reaction. One example could be the relationship between ice cream sales and drowning incidents. During summer, higher temperatures drive both trends: people buy more ice cream and engage in water activities, increasing drowning risks. Granger Causality might falsely suggest that ice cream sales predict drowning incidents because there is some delay between the two events, but the true cause is the confounding factor—temperature.

The presence of a so-called ‘unit root’ in a time series increases the risk of Type I errors (i.e., the risk of a false positive) [45]. A **unit root** is the term for an overall upward or downward trend over time. To mitigate this effect, the time series can be detrended by differencing, i.e., by calculating the difference between consecutive data points [23]. Essentially, detrending means that the underlying trend is removed from a time series, leaving behind the fluctuations or variations that are not explained by the trend. The goal of detrending is to focus on the remaining patterns or residuals, which may contain important information about short-term behavior or irregularities in the data. However, this detrending process may lead to information loss, particularly in data-sparse time series, which are often encountered in historical linguistics. We tried to determine whether there was a unit root in the time series using the Augmented Dickey–Fuller Test [46]. The results of the test will be discussed below per corpus, together with the results of the Granger Causality test.

Another cause of Type I errors is a greater temporal lag between two time series. As said, Granger Causality essentially compares two regression models: a model with only the autoregression of time series A, where the outcome y at time step t is predicted by its past values, together with an error term ε (1); and a model where y is predicted by the autoregression of time series A and the past values of time series B (the term $\sum_{i=1}^{k}\beta_{i}x_{t-i}$) (2). A Chi square test or an F-test can be used on the residual deviance to see whether the two regression models differ significantly [47,48]. Under the null hypothesis, all the β terms approach 0, which means that the models are not significantly different. The null hypothesis can be rejected when model (2) outperforms model (1), which means that time series A is Granger-caused by time series B. The result of the F-test depends on the number of time lags k (i.e., the number of time steps the model uses to look back) used. The higher the number of lags, the more β factors there are that can spuriously differ from 0. The higher the lag between the two time series, the greater the risk of Type I errors. To mitigate this risk, it is best to keep the number of lags low [49]. In the case at hand, we restricted ourselves to lags 1 to 10.

(1) $$y_{t}=\alpha_{0}+\sum_{i=1}^{k}\alpha_{i}y_{t-i}+\varepsilon_{t}$$

(2) $$y_{t}=\alpha_{0}+\sum_{i=1}^{k}\alpha_{i}y_{t-i}+\sum_{i=1}^{k}\beta_{i}x_{t-i}+\varepsilon_{t}$$

It is important to test the causation in two ways: (i) do changes in morphological complexity Granger-cause changes in word order rigidity, or (ii) do changes in word order rigidity Granger-cause changes in morphological complexity? We hypothesize that the causal relation runs from morphological complexity to word order rigidity and not the other way around, following the most common hypothesis that word order rigidity ‘compensates’ for the loss in inflectional morphology. Accordingly, in test (i), the difference between the two regression models has to be significant, and in test (ii), the difference between the two regression models has to be non-significant.

### 2.3. Corpora

Both Kolmogorov complexity and Granger Causality have a set of requirements that influence the selection of the data. The ideal type of data for a Kolmogorov complexity analysis is parallel data, which ensures that topic and orthography have a minimal influence on the complexity measure. However, there are very few parallel, diachronic corpora that allow us to look at diachronic change. While parallel data are ideal, it is not altogether impossible to obtain meaningful results with semi-parallel or even comparable data (see Ehret (2017: pp. 131–159) [26] for an application of Kolmogorov complexity to British English registers and English learner corpora). Since Kolmogorov complexity relies on the compression of reoccurring patterns, a second requirement is that the input data need to be large enough to ensure that such reoccurring patterns are indeed present. Additionally, when using semi-parallel or comparable data, all the texts in the input need to be the same size. Following [26,40], we define texts as having the ‘same size’ if they have the same number of sentences. We adhered to this principal corpus-internally; i.e., per corpus, we sampled an equal number of sentences for each year. However, this number was not kept equal across the different corpora. For each corpus, we selected as much data per year as was available in the year with the least data. Accordingly, we did not draw conclusions across the different corpora or languages but only answered the research question for each individual corpus, so that each corpus independently contributes evidence for the hypotheses. Some corpora contained a lot of data, even in the year with the least data, but sampling too many sentences was also not ideal, as this would have increased computation time. In practice, we took samples ranging from 1500 sentences (for DTA) to 5000 sentences (for C-CLAMP).

Granger Causality works with time series data. A time series is a sequence of data points collected or recorded at successive, evenly spaced time intervals. The two main requirements for this technique are that the time series is uninterrupted and has sufficient length. Interruptions or gaps in the time series data may distort or obscure the true causal relationships between variables, leading to inaccurate results. Granger Causality analysis relies on the concept of time lags, where the past values of one variable are used to predict the future values of another variable. If the time series data are interrupted, the temporal sequence required for accurate time lag analysis is disrupted as well. A sufficient length of time series data is required to establish meaningful relationships between variables.

Selecting corpora and sampling is thus a difficult balancing act. We selected diachronic corpora containing as much data as possible over a sufficiently long time span (required for Granger Causality), while aiming for homogeneity (required for Kolmogorov complexity) within each corpus. Some of the corpora did not have enough data for an analysis per year, so for these, we opted for an analysis per decade instead. We created a random sample per time unit for the analysis. Table 1 gives an overview of the corpora, the level of granularity (year or decade), the sample size, the time span, and the genre of the corpus.

### 2.4. Measuring Morphological Complexity and Word Order Rigidity

The objective of this study was to investigate whether changes in morphological complexity can predict changes in word order rigidity, as a proxy for syntactic complexity. To achieve this, we needed to calculate the morphological complexity ratio and word order rigidity ratio. However, simply applying compression to the entire text was insufficient, since we wished to analyze morphological complexity and word order rigidity separately. Instead, we had to modify the text at the morphological and syntactic level before compression. The complexity ratios were determined by dividing the file size of the compressed and distorted files (resulting from steps 2 and 3 below) by the compressed file without distortion (resulting from step 1) [26,38]. To be more specific, the following steps were taken:

1. Compress the original file using Gzip.
2. Introduce distortion to the original file, creating the distorted file.
3. Compress the distorted file once again using Gzip.
4. Calculate the complexity ratio.

**Morphological distortion** is implemented by randomly removing 10% of the orthographically transcribed characters in the text. This alteration disrupts the information at the morphological level and compromises morphological regularity, affecting inflectional endings (e.g., *giving* can become *givig*) and roots and stems (e.g., *fossil* can become *fssil*). The random removal of characters essentially gives rise to new word forms generated haphazardly, which means that the number of unique tokens in the text increases. The distortion will have a smaller effect on the compression of complex texts with a high surface token diversity, because they already contain a higher number of unique tokens before distortion. The effect of the distortion on the compression of less complex texts will be comparatively larger, because these texts have a low surface token diversity before distortion, but their diversity increases remarkably after distortion.

**Syntactic distortion** happens in the same way as morphological distortion, but instead of characters, words are removed. This process disrupts the word order rules (e.g., a 4-gram such as *it should have been* can become *it should been*), resulting in a greater number of unique n-grams. Consequently, the compressibility of the text is adversely affected, leading to decreased compression performance. Complex texts, with strict word order, will be more affected by distortion, because they contain more structural surface redundancies. In other words, complex texts have more recurring n-grams that can be jumbled during distortion. Syntactically simple texts with free word order already have fewer structural surface redundancies and will therefore be less affected by the distortion (thus facilitating compressibility).

The distortion method employed implies that the syntactic complexity ratio reflects the rigidity of the word order, rather than providing a comprehensive representation of the overall syntactic complexity of a language. While it is true that word order is not the exclusive means by which languages convey functions or meanings through syntactic strategies, it is a strategy at the opposite end from affixal morphology on the morphology–syntax cline. Additional strategies encompass semi-morphological aspects such as agreement and cross-reference. With this approach, we followed Bakker (1998) [56], who also investigated syntactic complexity on the basis of word order rigidity or ‘flexibility’, where flexible languages are syntactically simple and inflexible languages are syntactically complex. From the perspective of language processing (rooted within relative complexity), rigid word order is normally defined as syntactically simple, because strict word order is easier to process than free word order. The methodology of the research at hand is embedded within the absolute view on complexity, which focusses on the number of word order rules and constraints. In this context, a language with strict word order is considered more complex, because it imposes more restrictions or constraints on the rules governing word order. This idea of complexity as constraints goes back at least to Greenberg (1960) [57], who calculated the proportion of word order links over the total number of ‘nexus’ (Greenberg’s term for grammatical relations). To avoid confusion, we will henceforth talk about word order rigidity instead of syntactic complexity.

The morphological complexity ratio and the word order rigidity ratio were calculated as follows:

(3) $$morphological\;complexity\;ratio=-\frac{compressed\;file\;size\;after\;morphological\;distortion}{compressed\;file\;size}$$

(4) $$word\;order\;rigidity\;ratio=\frac{compressed\;file\;size\;after\;syntactic\;distortion}{compressed\;file\;size}$$

It is important to observe that the word order rigidity ratio is represented by a positive value, while the morphological complexity ratio is indicated by a negative value. This distinction follows from the nature of the distortion process employed. In the case of morphological distortion, we anticipated that a complex text would be less affected, resulting in a negative value. Conversely, with syntactic distortion, we expected a complex text to be more impacted, leading to a positive value. Making the morphological complexity ratio negative and the syntactic complexity ratio positive ensured easier interpretation of the results. Indeed, in both ratios, higher values (marked by a smaller difference between numerator and denominator) indicated greater complexity.

The use of random deletion means that it is in theory possible that similar types of elements are repeatedly deleted in a particular text. For example, with regard to morphological distortion, it is possible that in a text, only roots or only affixes are deleted. With regard to syntactic distortion, it is possible that the same words are deleted, while others are left untouched. In order to mitigate this aleatoric effect of the randomization in our study, for each text, the morphological complexity ratio and word order rigidity ratio were computed over 100 iterations, whose means were then calculated. In other words, for each text, the mean morphological complexity ratio and mean word order rigidity ratio were retained.

Finally, we had to turn these complexity measures into two time series: one for morphology and one for syntax (word order rigidity). Importantly, a time series requires a measurement at each time step; in other words, it is not allowed to have gaps. If we were to have created a time series with time steps of one year, such gaps would have presented a problem. Accordingly, we operated with time steps of one decade rather than one year. For each of these time steps, we calculated the mean morphological complexity ratio and the mean word order rigidity ratio. These means make up our time series.

## 3. Analysis and Results

For each corpus, we examined whether there is a causal relationship between morphological complexity and word order rigidity. First, a covariate-augmented Dickey–Fuller test [58] was applied to the time series to check for a unit root (see Section 2.2). Table 2 gives an overview of the results of the test. A significant result (*p* < 0.05), indicated in bold, means that the time series does not contain a unit root and detrending is not necessary. However, it is recommended that the other time series, which do contain a unit root, are detrended. It is not possible to compare a detrended time series with one that has not been detrended, because a detrended time series is one time step shorter than its original. This is why we applied Granger Causality to both the original and the detrended time series in both directions (i.e., from morphology to word order and the other way around). We examined the relationship between morphology and word order up to 10 time steps (lags) back in time. For corpora with yearly time series data, we used a lag of up to 10 years. However, time series from corpora with data measured per decade were shorter, so fewer lags were preferable to ensure accurate analysis. Thus, for CLMET and Frantext, we opted for 5 lags.

**Figure 1:**
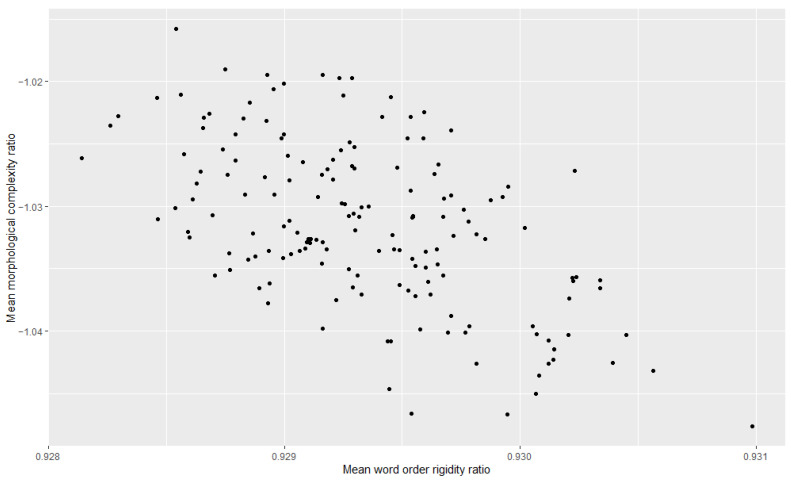
Visualization of the linear regression model; scatterplot of the morphological complexity and word order rigidity measures per time step in C-CLAMP.

**Figure 2:**
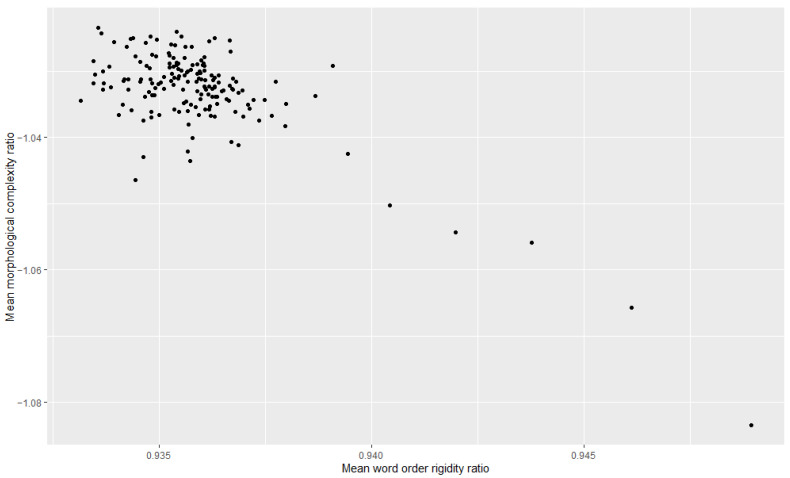
Visualization of the linear regression model; scatterplot of the morphological complexity and word order rigidity measures per time step in COHA.

**Figure 3:**
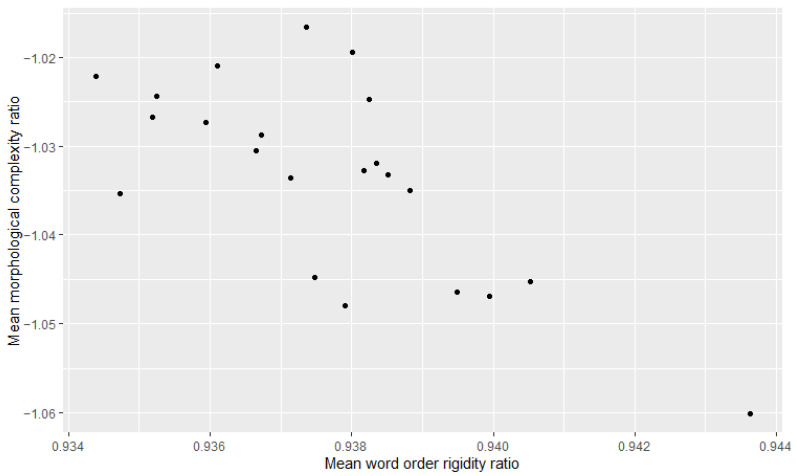
Visualization of the linear regression model; scatterplot of the morphological complexity and word order rigidity measures per time step in CLMET.

**Figure 4:**
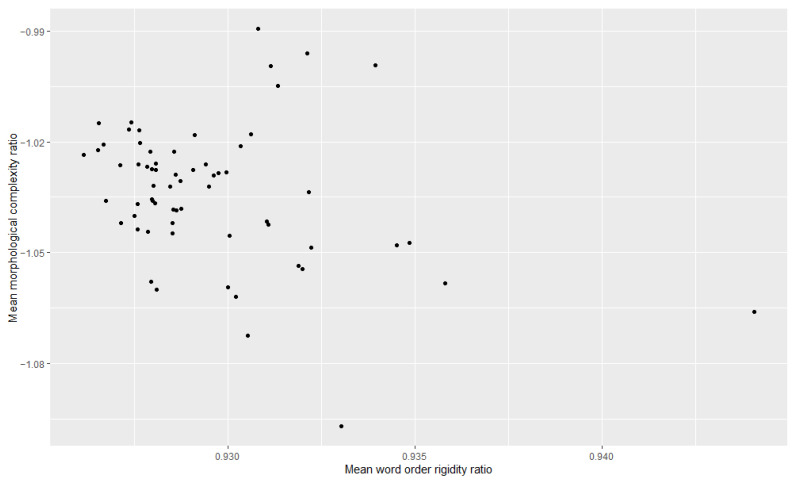
Visualization of the linear regression model; scatterplot of the morphological complexity and word order rigidity measures per time step in DTA.

**Figure 5:**
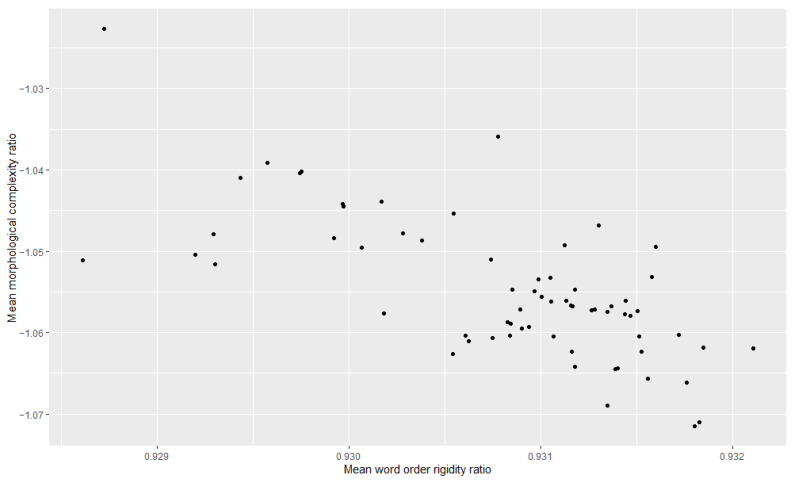
Visualization of the linear regression model; scatterplot of the morphological complexity and word order rigidity measures per time step in L’Unità corpus.

**Figure 6:**
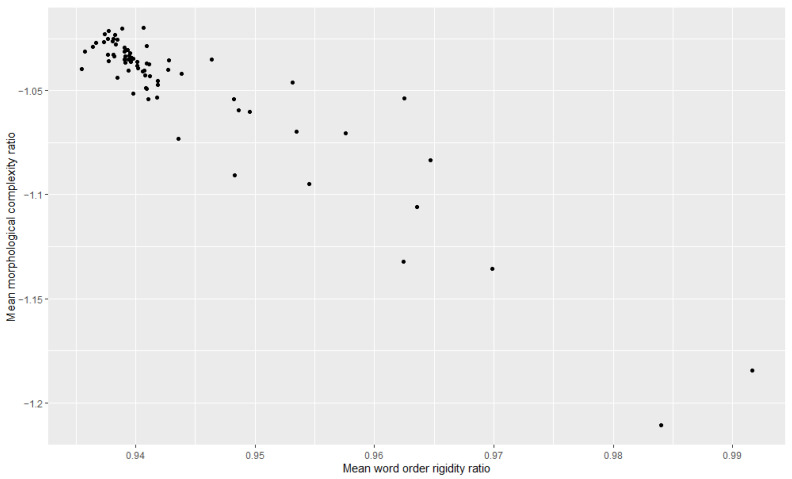
Visualization of the linear regression model; scatterplot of the morphological complexity and word order rigidity measures per time step in Frantext.

Additionally, we examined the relationship between morphological complexity and word order rigidity through linear regression, to see whether there is indeed a trade-off; i.e., we tried to see if years/decades with high morphological complexity have low word order rigidity and vice versa. The results can be appreciated in Table 3, Table 4, Table 5, Table 6, Table 7 and Table 8 and Figure 1, Figure 2, Figure 3, Figure 4, Figure 5 and Figure 6. Each corpus produced the same results: there is indeed a significant negative correlation between morphological complexity and word order rigidity.

**Figure 7:**
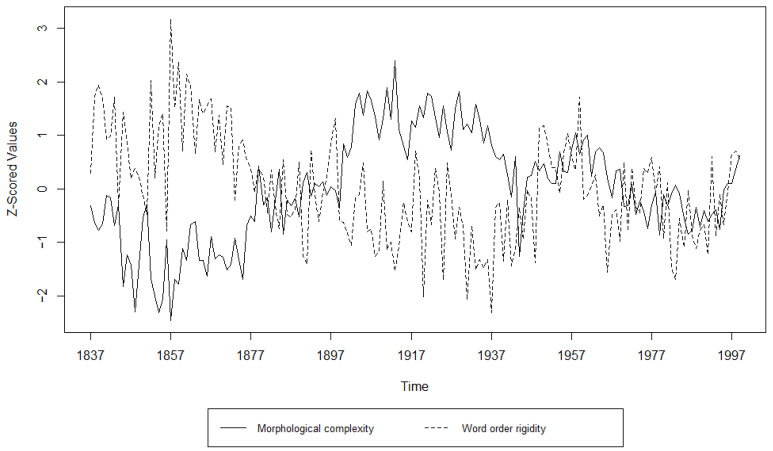
Visualization of the Granger Causality analysis; z-scored morphological complexity and word order rigidity measures per year in C-CLAMP.

**Figure 8:**
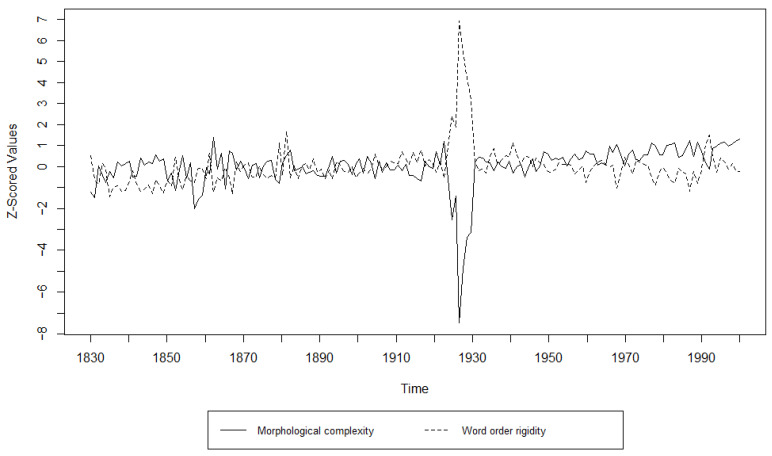
Visualization of the Granger Causality analysis; z-scored morphological complexity and word order rigidity measures per year in COHA.

**Figure 9:**
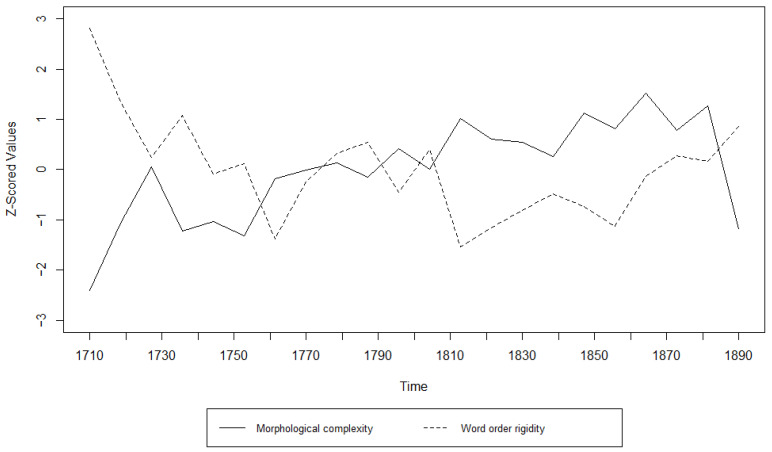
Visualization of the Granger Causality analysis; z-scored morphological complexity and word order rigidity measures per year in CLMET.

**Figure 10:**
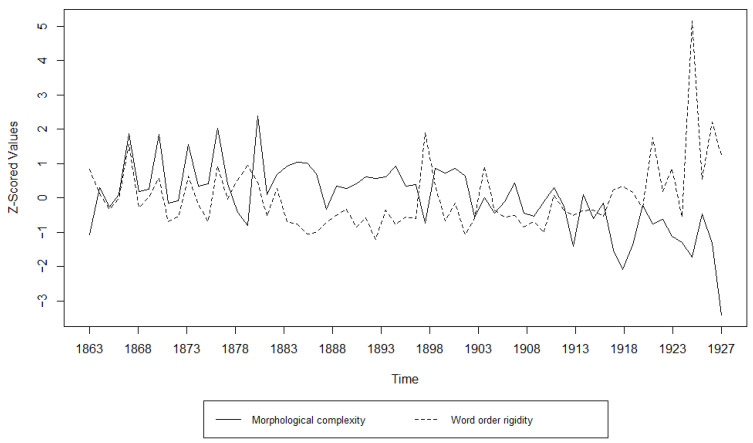
Visualization of the Granger Causality analysis; z-scored morphological complexity and word order rigidity measures per year in DTA.

**Figure 11:**
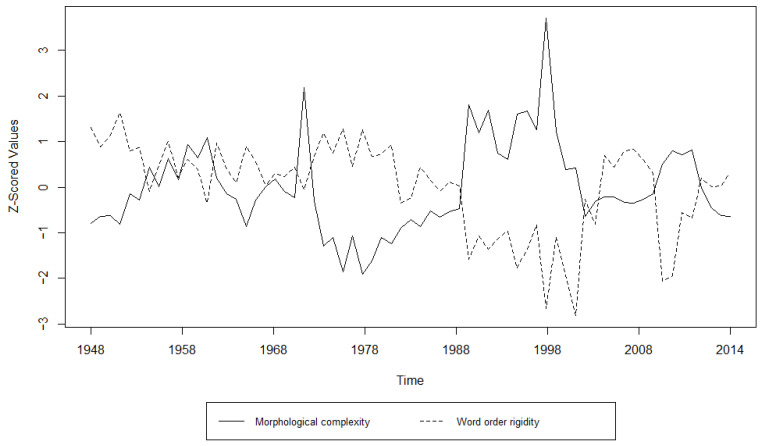
Visualization of the Granger Causality analysis; z-scored morphological complexity and word order rigidity measures per year in L’Unità corpus.

**Figure 12:**
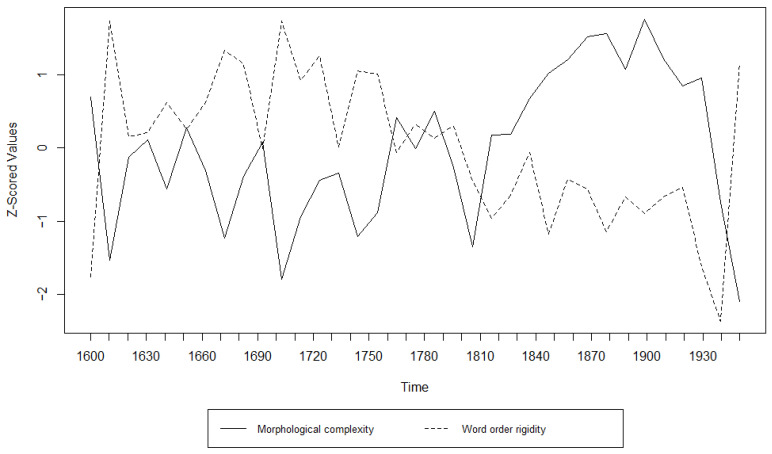
Visualization of the Granger Causality analysis; z-scored morphological complexity and word order rigidity measures per decade in Frantext.

Turning to the Granger Causality analysis, the results can be found in Table 9, Table 10, Table 11, Table 12, Table 13 and Table 14 and Figure 7, Figure 8, Figure 9, Figure 10, Figure 11 and Figure 12. The results for the Germanic languages, except for the CLMET corpus, show that changes in morphological complexity indeed Granger-cause changes in word order rigidity if the time series are not detrended. The only exception is the COHA corpus, where changes in morphological complexity can still be said to Granger-cause changes in word order rigidity even when the time series have been detrended. Most of the Germanic corpora show significance at several lags, which indicates that the results are less likely to be spurious. C-CLAMP and DTA both show significance at the lower time steps, lags 1–2 and lags 1–4, respectively. While COHA shows significance at the higher lags of 4–10, CLMET is the only Germanic corpus that shows no significant results in either direction.

The results for the Romance languages, however, do not weigh in favorably for a causal relation. The Italian corpus shows no significant effects in either direction, with or without the detrending of the time series. The French corpus shows significant effects in both directions at lag 1 with and without detrending. Since causation can only run in one direction, we have to reject this result. At higher lags (lags 2, 3, and 5 without detrending and lags 2 and 3 with detrending), we found a significant (*p* < 0.05) causal relation running from word order rigidity to morphological complexity. This means that, strictly speaking, in Frantext, changes in word order rigidity Granger-cause changes in morphological complexity at these higher lags. This result is dubious, given the bidirectional ‘causation’ at lag 1.

## 4. Discussion and Conclusions

In this study, we set out to examine the relationship between morphological complexity and word order rigidity in terms of causality. Whether changes in morphology drive changes in word order or the other way around is still a much debated moot point within linguistics [17,18,19,20]. The most commonly held hypothesis with the most substantial quantitative evidence is that morphological changes typically precede shifts in word order [12,23,24]. However, the question of whether this hypothesis holds true in relation to linguistic complexity has not been thoroughly investigated to date. We have tried to answer this question using both Kolmogorov complexity as a ‘holistic’ (i.e., aggregate) measure of morphological complexity and word order rigidity and Granger Causality as a means of identifying causation.

For the Germanic languages in our sample, the aforementioned hypothesis was borne out in all but one corpus. Remarkably, the corpus where the model fails to detect the expected Granger Causality is the only one with a lower-resolution measurement, at the level of decades rather than years. It would appear, then, that the method requires enough variability and enough measurement points. The anomaly could be an artefact of the data, though we do not want to overstate our claims and must retain some caution.

The low-resolution problem may also explain why the method fails to give robust results for the Romance languages in our sample: the French corpus has time measured in decades, while the Italian corpus has time measured in years but covers only half a century. This suggests that we are in urgent need of better corpora in historical linguistics.

The difference in the results displayed by the Germanic and the Romance languages could also have a linguistic explanation. One possibility is that the Germanic languages simply exhibit more outspoken differences in complexity over time. Note that overall, the timespans which we examined are not very extensive. Deflection is a process that takes several centuries. It is not unlikely, therefore, that it does not (yet) clearly manifest itself in the short time spans covered by our corpora. In this sense, the Germanic languages may very well be the odd ones out, because they do show these differences, again over quite short time spans. Indeed, Germanic languages form a branch of the Indo-European languages that is known for its rampant reduction in morphological complexity [2]—though it is generally held that German holds out better, at least in the written language, with numerous inflection intricacies that are unknown to its large West Germanic siblings and even to its Romance cousins like French and Italian.

Another explanation may be found in the system of clitic pronouns in the Romance languages. Clitic pronouns cannot be considered fully independent words and thus should not be considered completely analytical from the point of view of morphological typology. Furthermore, they exhibit highly variable behavior in the Romance languages (Pescarini 2021) [59]. It is a possibility that systems of clitic pronouns may blur the analysis. However, Ehret (2017) [26] has conducted a similar analysis; i.e., she tested Kolmogorov complexity on a synchronic corpus including both Romance and Germanic languages, where linguistically sensible results were obtained, indicating that the analysis is robust.

Even so, the results point to an asymmetry, which can best be stated in conditional form: if there is a significant Granger Causality to be detected in a language (which may depend on its time resolution), then it is never the case that word order rigidity Granger-causes changes in morphological complexity, whereas it is common to find morphological complexity Granger-causing changes in word order rigidity. This tentatively supports the word-order-as-a-compensation strategy for the West European languages, confirming findings from earlier studies [7,8,9,10,11,12]. This study can also be compared to other works that use Kolmogorov complexity to gain insight into linguistic complexity [26,36,38,39]. The trade-offs that we observe within each language are particularly similar to those identified between languages in the studies by Ehret (2017) [26] and Ehret and Szmrecsanyi (2016) [40].

Importantly, the results work best when the time series have not been detrended. Detrending a time series involves removing trends, i.e., long-term systematic changes, from the data to focus on the more immediate or cyclical behaviors. However, in linguistics, these long-term systematic changes are exactly what is of interest in the data. Deflection is a long-term diachronic process that spans several centuries [2,5]. It could thus be argued that with linguistic phenomena, and especially given the specific research question that we are interested in, detrending may be unnecessary or even obfuscating.

## Figures and Tables

**Table 1:** Overview of the corpora.

Corpus	Language	Year/Decade	Sample Size (Sentences)	Time Span	Genre
C-CLAMP [50]	Dutch	Year	5000	1837–1999	Cultural and literary journals
The Corpus of Historical American English (COHA) [51]	American English	Year	4000	1830–1999	Only the fiction texts are taken into account
The Corpus of Late Modern English Texts, version 3.0 (CLMET3.0) [52]	British English	Decade	2000	1710–1920	Narrative fiction, narrative non-fiction, drama, letters, and treatise
Deutsches Textarchiv (DTA) [53]	German	Year	1500	1863–1927	Not genre-consistent
L’Unità [54]	Italian	Year	5000	1948–2014	Newspapers
Frantext [55]	French	Decade	5000	1600–1940	Not genre-consistent

**Table 2:** *p*-values of the covariate-augmented Dickey–Fuller test; significant results at an α level of 0.05.

	CCLAMP	COHA	CLMET	DTA	L’Unità	Frantext
Morphology	0.2916	**0.001219**	0.6638	**0.0002868**	0.2934	0.8443
Word order	**0.0005695**	**0.004377**	0.5149	0.06443	0.2232	**0.04433**

**Table 3:** Linear regression model of morphological complexity over word order rigidity for C-CLAMP (adjusted R-squared = 0.313).

	Estimate	Std. Error	t Value	Pr (>|t|)
Intercept	5.6206	0.7691	7.308	<0.0001
Word order rigidity	−7.1578	0.8275	−8.650	<0.0001

**Table 4:** Linear regression model of morphological complexity over word order rigidity for COHA (adjusted R-squared = 0.5626).

	Estimate	Std. Error	t Value	Pr (>|t|)
Intercept	1.5096	0.1720	8.774	<0.0001
Word order rigidity	−2.7166	0.1838	−14.778	<0.0001

**Table 5:** Linear regression model of morphological complexity over word order rigidity for CLMET 3.0 (adjusted R-squared = 0.4795).

	Estimate	Std. Error	t Value	Pr (>|t|)
Intercept	2.4469	0.7716	3.171	<0.0001
Word order rigidity	−3.7117	0.8229	−4.510	<0.0001

**Table 6:** Linear regression model of morphological complexity over word order rigidity for DTA (adjusted R-squared = 0.08424).

	Estimate	Std. Error	t Value	Pr (>|t|)
Intercept	0.4010	0.3980	1.008	0.3155
Word order rigidity	−1.5496	0.4274	−3.625	<0.0001

**Table 7:** Linear regression model of morphological complexity over word order rigidity for L’Unità corpus (adjusted R-squared = 0.4864).

	Estimate	Std. Error	t Value	Pr (>|t|)
Intercept	6.1967	0.9100	6.810	<0.0001
Word order rigidity	−7.7905	0.9776	−7.969	<0.0001

**Table 8:** Linear regression model of morphological complexity over word order rigidity for Frantext (adjusted R-squared = 0.8519).

	Estimate	Std. Error	t Value	Pr (>|t|)
Intercept	1.7899	0.1423	12.58	<0.0001
Word order rigidity	−3.0067	0.1507	−19.95	<0.0001

**Table 9:** *p*-values of the Granger Causality analysis per lag with and without detrending for C-CLAMP.

	Without Detrending		With Detrending	
Lag	Morphology Causes Word Order	Word Order Causes Morphology	Morphology Causes Word Order	Word Order Causes Morphology
1	**0.001402**	0.1755	0.4752	0.5094
2	**0.02456**	0.5463	0.7249	0.1887
3	0.07971	0.3151	0.8092	0.2566
4	0.06928	0.3661	0.8956	0.3564
5	0.1328	0.4958	0.9473	0.3461
6	0.1856	0.4948	0.9623	0.3843
7	0.1364	0.4516	0.8261	0.4534
8	0.195	0.5532	0.8464	0.2641
9	0.2664	0.3497	0.831	0.3763
10	0.248	0.4806	0.8451	0.3036

**Table 10:** *p*-values of the Granger Causality analysis per lag with and without detrending for COHA.

	Without Detrending		With Detrending	
Lag	Morphology Causes Word Order	Word Order Causes Morphology	Morphology Causes Word Order	Word Order Causes Morphology
1	0.858	0.8623	0.1816	0.8783
2	0.4833	0.7976	0.1682	0.9423
3	0.3735	0.9036	**0.00112**	0.9318
4	**0.0005979**	0.8865	**0.001819**	0.9047
5	**0.001591**	0.8979	**0.005217**	0.8006
6	**0.009824**	0.852	**0.004183**	0.7589
7	**0.007966**	0.8743	**0.009972**	0.8227
8	**0.0139**	0.9062	**0.01866**	0.8045
9	**0.02486**	0.8858	**0.02058**	0.941
10	**0.03655**	0.9473	**0.01578**	0.8172

**Table 11:** *p*-values of the Granger Causality analysis per lag with and without detrending for CLMET 3.0.

	Without Detrending		With Detrending	
Lag	Morphology Causes Word Order	Word Order Causes Morphology	Morphology Causes Word Order	Word Order Causes Morphology
1	0.2877	0.5308	0.2774	0.2774
2	0.2544	0.6607	0.1641	0.1641
3	0.3219	0.08349	0.2621	0.2621
4	0.4308	0.3023	0.3442	0.3442
5	0.68	0.3935	0.3706	0.3706

**Table 12:** *p*-values of the Granger Causality analysis per lag with and without detrending for DTA.

	Without Detrending		With Detrending	
Lag	Morphology Causes Word Order	Word Order Causes Morphology	Morphology Causes Word Order	Word Order Causes Morphology
1	**0.005058**	0.35	0.2139	0.3681
2	**0.01473**	0.3295	0.05311	0.8995
3	**0.03366**	0.9487	0.07508	0.9928
4	**0.01518**	0.9929	0.05801	0.71
5	0.05535	0.8028	0.2451	0.9412
6	0.1125	0.9778	0.3804	0.9212
7	0.1532	0.953	0.3523	0.7832
8	0.08149	0.8473	0.2842	0.8567
9	0.09821	0.9037	0.317	0.9328
10	0.05143	0.9121	0.2332	0.8846

**Table 13:** *p*-values of the Granger Causality analysis per lag with and without detrending for L’Unità corpus.

	Without Detrending		With Detrending	
Lag	Morphology Causes Word Order	Word Order Causes Morphology	Morphology Causes Word Order	Word Order Causes Morphology
1	0.734	0.7296	0.2733	0.8145
2	0.3631	0.8849	0.5469	0.8706
3	0.5807	0.9053	0.338	0.8134
4	0.475	0.8846	0.4782	0.9604
5	0.6295	0.9537	0.5042	0.9769
6	0.6002	0.975	0.4484	0.9221
7	0.5087	0.9233	0.4815	0.9574
8	0.475	0.9689	0.587	0.9481
9	0.5008	0.9369	0.6787	0.934
10	0.5579	0.8835	0.8587	0.9117

**Table 14:** *p*-values of the Granger Causality analysis per lag with and without detrending for Frantext.

	Without Detrending		With Detrending	
Lag	Morphology Causes Word Order	Word Order Causes Morphology	Morphology Causes Word Order	Word Order Causes Morphology
1	**0.01698**	**0.02445**	**0.01689**	**0.007708**
2	0.05865	**0.02889**	0.188	**0.006095**
3	0.1806	**0.01083**	0.6095	**0.04238**
4	0.6813	0.0687	0.6362	0.06929
5	0.7898	**0.04298**	0.6639	0.05629

## Data Availability

The data sets and scripts are available at https://zenodo.org/records/14162717 (accessed on 14 November 2024). The samples of the corpora are available at https://zenodo.org/records/14162733 (accessed on 14 November 2024).
